# Supplementary material for: Superconductivity above 200 K discovered in superhydrides of calcium
Source: Nat Commun. 2022 May 23;13:2863. doi: 10.1038/s41467-022-30454-w (PMC9126910; doi:10.1038/s41467-022-30454-w)
Supplement: Supplementary file 1 — Supplementary Information [file 41467_2022_30454_MOESM1_ESM.pdf]

Supplementary Materials

Superconductivity above 200 K Discovered in Superhydrides of Calcium

Zhiwen Li [a1,2], Xin He [a1,2,3], Changling Zhang[a1,2], Xiancheng Wang*[1,2], Sijia Zhang[1], Yating Jia[1], Shaomin Feng[1], Ke Lu[1,2], Jianfa Zhao[1,2], Jun Zhang[1,2], Baosen Min[1,2], Youwen Long[1,2,3], Richeng Yu[1,2], Luhong Wang[4], Meiyan Ye[5], Zhanshuo Zhang[5], Vitali Prakapenka[6], Stella Chariton[6], Paul A. Ginsberg[7], Jay Bass[7], Shuhua Yuan [8], Haozhe Liu[8], Changqing Jin*[1,2,3]

[1] *Beijing National Laboratory for Condensed Matter Physics, Institute of Physics, Chinese Academy of Sciences, Beijing 100190, China*

[2] *School of Physical Sciences, University of Chinese Academy of Sciences, Beijing 100190, China*

[3] *Songshan Lake Materials Laboratory, Dongguan 523808, China*

[4] *Harbin Institute of Technology, Harbin 130001, China*

[5] *Jilin University, Changchun, China*

[6]*Center for Advanced Radiations Sources, University of Chicago, Chicago, Illinois 60637, USA*

[7]*Department of Geology, University of Illinois at Urbana-Champaign, Urbana, Illinois, 61801, USA*

[8] *Center for High Pressure Science & Technology Advanced Research, Beijing 100094, China*

[a] Contributed Equally. *The Corresponding Authors: Wangxiancheng@iphy.ac.cn; Jin@iphy.ac.cn

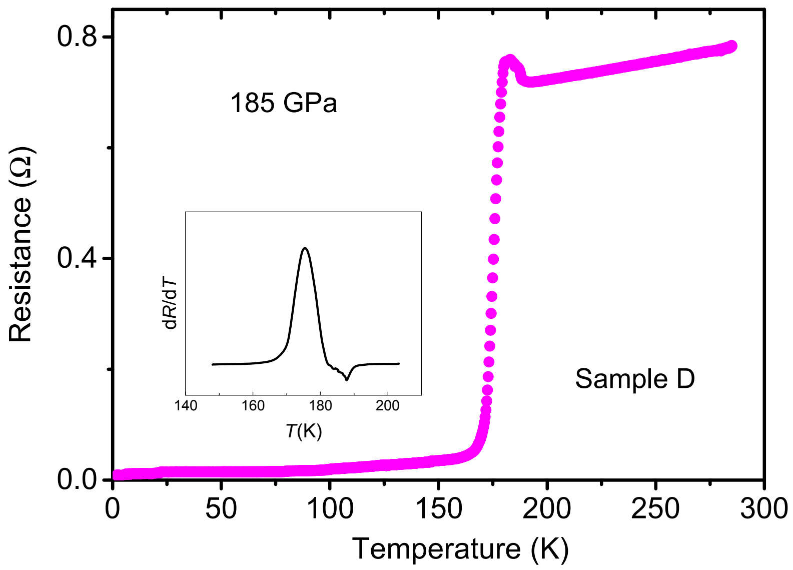

Supplementary Fig. 1: The superconducting transition of a calcium superhydride. The temperature dependence of resistance measured for Sample D at 185 GPa. The inset is the enlarged view of the derivative of resistance over temperature (dR/dT).

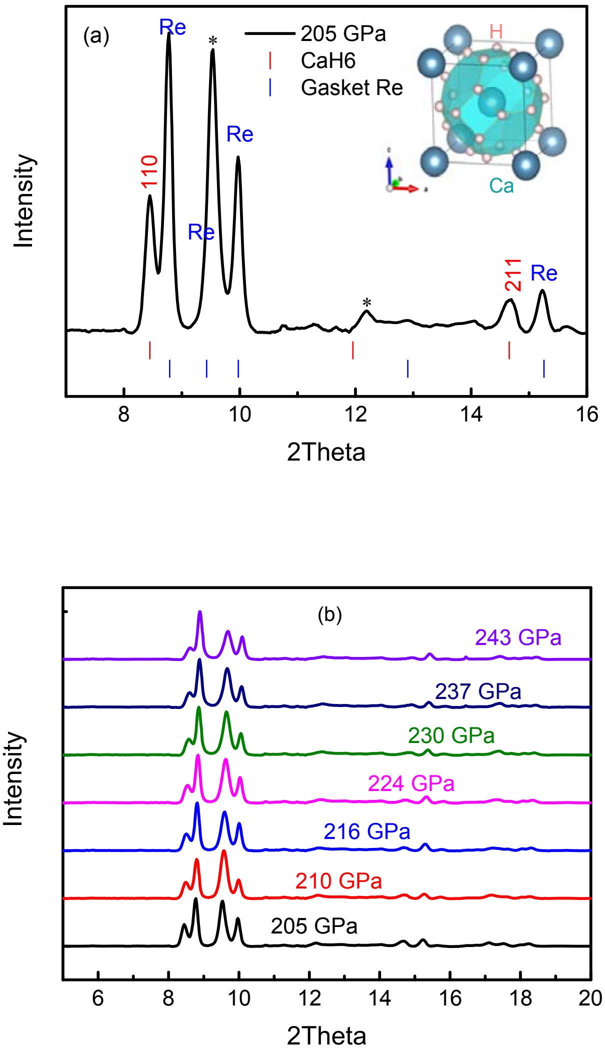

Supplementary Fig. 2: The structure characterizations. (a) The high pressure synchrotron X-ray diffraction pattern measured at room temperature of one synthesized sample, which shows the presence of $Im\overline{3}m$ CaH_6 with a=3.21 Å. The inset is the sketch of crystal structure of $Im\overline{3}m$ CaH_6. Re is the gasket to hold the sample in high pressure. The * denotes the unknown peaks. (b) The X-ray diffraction patterns measured at different high pressures.
